# Persistent articular infection and host reactive response contribute to *Brucella*-induced spondyloarthritis in SKG mice

**DOI:** 10.1128/mbio.00542-25

**Published:** 2025-05-14

**Authors:** Jerome S. Harms, Michael Lasarev, Thomas Warner, Sergio Costa Oliveira, Judith A. Smith

**Affiliations:** 1 Departments of Pediatrics and Medical Microbiology and Immunology, University of Wisconsin-Madison 5228, Madison, Wisconsin, USA; 2 Department of Biostatistics and Medical Informatics, University of Wisconsin-Madison 198622, Madison, Wisconsin, USA; 3 Department of Pathology and Laboratory Medicine, University of Wisconsin-Madison 189586, Madison, Wisconsin, USA; 4 Departamento de Imunologia, Instituto de Ciências Biomédicas, Universidade de São Paulo 28133, São Paulo, São Paulo, Brazil; Washington University in St. Louis, St. Louis, Missouri, USA; University of California Davis, Davis, California, USA

**Keywords:** Brucella, mouse model, spondyloarthritis

## Abstract

Brucellosis, one of the most prevalent zoonotic diseases worldwide, often results in osteoarticular complications including large joint and axial arthritis mimicking spondyloarthritis. To model this chronic manifestation, we infected autoimmunity-prone SKG mice containing a mutation in the T cell adaptor ZAP-70 with *Brucella* species. *B. melitensis* infection resulted in a fully penetrant, readily scoreable disease involving large joint wrist and foot arthritis, peri-ocular inflammation, and less frequent scaly paw rash. Infection with *B. abortus* resulted in similar manifestations, although with delayed arthritis onset, and *B. neotomae* revealed sex differences, with a more severe disease in females. Heat-killed *Brucella* did not induce arthritis, evincing a requirement for viable infection. Across species, splenic CFU correlated well with final clinical score at 12 weeks (ρ = 0.79 and *P* < 0.001). Moreover, viable *Brucella* was recovered from the paws at 12 weeks, consistent with persistent articular infection. Mice infected with a *Brucella*Δ*tcpB* mutant lacking a Type IV secretion system-dependent mediator displayed an intermediate phenotype without significant differences in splenic CFU. Thus, the degree of arthritis did not strictly correlate with the degree of systemic infection, but it suggested an additional reactive component. Together, these data suggest that *Brucella*-induced spondyloarthritis reflects both persistent colonization and excess host reactivity. Moreover, the sensitivity of the SKG model to different species and mutants will provide new opportunities for dissecting correlates of *Brucella* virulence and host immunity.

**IMPORTANCE:**

Brucellosis, a bacterial infection acquired from herd animals, remains one of the most common zoonotic diseases worldwide. Chronic infection often results in spondyloarthritis-like complications. Investigation into pathogenesis has been limited by the lack of overt disease in standard laboratory mice. We addressed this issue using spondyloarthritis-susceptible SKG mice. Upon infection with *B. melitensis*, SKG mice develop robust, fully penetrant large joint arthritis. Arthritis development required viable bacteria, and live *Brucella* persisted in paw tissue out to 12 weeks. Disease onset, severity, and manifestations varied upon infection with different *Brucella* species and mutants, suggesting an additional immune reactive component. Together, these results suggest that this new model will be very useful to the scientific community for determining correlates of bacterial virulence leading to clinical disease.

## INTRODUCTION

Brucellosis, caused by the facultative intracellular bacteria *Brucella* species, remains one of the most prevalent zoonosis worldwide, afflicting people in South America, Mexico, Europe (Mediterranean basin), the Middle East, Africa, and Asia (1, 2). Initially, infection manifests with undulant fever, myalgias, and arthralgias. However, chronic brucellosis may affect many host organs, leading to arthritis, orchitis, liver damage, endocarditis, and encephalomyelitis (3, 4). Osteoarticular involvement (sacroiliitis, peripheral arthritis, spondylitis, osteomyelitis, and tenosynovitis) is the most frequently reported complication, affecting up to 20–87% of subjects (5, 6). Indeed, differentiation of chronic brucellosis from rheumatologic spondyloarthritis can be challenging in endemic areas. *B. melitensis*, which infects sheep or goats, is the primary human pathogen, although human infections with *B. abortus* (cows) predominates in certain locations, and *B. suis* (pigs) infections also occur (2, 7). Of these species, *B. melitensis* is considered the most virulent species in humans and associates with the more severe manifestations (2, 8). Brucellosis is challenging to treat, requiring prolonged courses of multiple antibiotics and relapses that occur in 5–10% of cases (3, 4).

The study of *Brucella* osteoarticular disease has been hampered by limitations in current mouse models. BALB/c and C57BL/6 mice, the most commonly used *Brucella* models, develop a transient spread of *Brucella* to their tails and paws 7–10 days post-infection (9). However, this colonization is mostly subclinical and requires high doses of luminescent *Brucella* and biophotonic imaging to detect (9–11). Chronically infected BALB/c females have been reported to develop peripheral reactive arthritis 26 weeks post-infection, but with extremely low penetrance (11). IFN-γ−/− mice develop arthritis after intra-peritoneal injection, but IFN-γ deficiency severely compromises *Brucella* containment (12). Similarly, the NOD-SCID model develops destructive tail osteomyelitis in response to the vaccine strain S19, but it completely lacks an adaptive immune system (13). Foot pad and joint injections of bacteria do not capture the systemic and chronic facets of disease (14).

To address these limitations, we turned to a murine model frequently used to investigate rheumatologic spondyloarthritis, the “SKG” mouse. Named for their discoverer (Shimon Sakaguchi), SKG mice bear a spontaneous mutation in the T cell receptor signaling adaptor ZAP-70 (W163C) that generates an autoimmunity-prone T cell repertoire. Mice under specific pathogen-free conditions do not develop disease without a trigger. Injection of the fungal cell wall component curdlan (β-1,3-glucan) results in enthesitis, peripheral and axial arthritis, vertebral inflammation, ileitis, and uveitis (15). The curdlan-injected mice develop a clinically obvious disease in a few weeks, with greater severity and rapidity in females. Intriguingly, this model is also dependent upon IL-23 and the presence of fecal microbiota (16, 17). SKG mice have also proven useful in modeling TNF-dependent *Chlamydia*-induced reactive arthritis (18). Finally, the SKG mice are on the BALB/c background, which is more susceptible to *Brucella* than other strains (19).

In this study, we investigated the clinical responses of SKG mice to different species and attenuated mutants of *Brucella*. Additionally, we sought to determine if the arthritis represented a purely “reactive” response to the initial infectious stimulus or persistent infection. Together, our results suggest that the severity of disease development reflects contributions both from persistent systemic infection and excess host reactivity. This new *Brucella* SKG model should prove highly useful for probing *in vivo* correlates of bacterial virulence and host immune responses to infection that result in spondyloarthritis manifestations.

## RESULTS

### *B. melitensis* induces spondyloarthritis-like clinical manifestations in SKG mice

**Fig 1:**
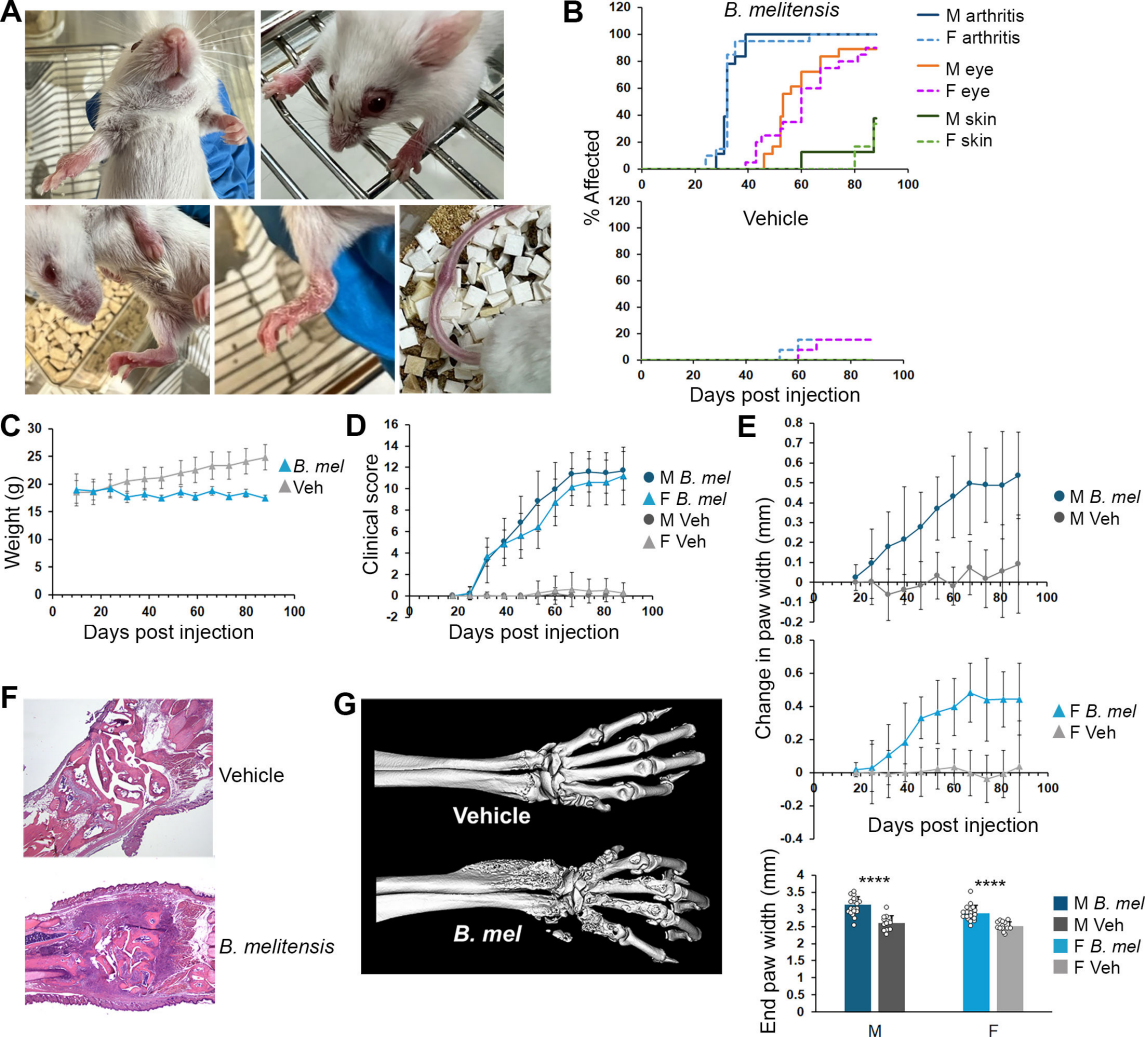
Clinical manifestations of *B. melitensis* infection in SKG mice. Mice were injected with PBS (vehicle control) or 2 × 10^6^ *B. melitensis* intra-peritoneally. Results are aggregates from at least three experiments and include 18 infected males (M), 12 control males, 19 infected females (F), and 13 control females. (**A**) Photographs of mice infected with *B. melitensi*s showing (top left then clockwise): front paw arthritis, peri-ocular inflammation, tail swelling, scaly rash, and mid-foot swelling. (**B**) Percent of mice developing arthritis, peri-ocular inflammation, and rash over time in male and female mice. Eye and skin disease occurred later than arthritis (*P* < 0.001). (C) Weights (grams) from infected or control female mice (*N* = 6 and 4 mice, respectively; *P* = 0.028 for weight loss in infected animals vs *P* < 0.001 for weight gain in controls; *P* < 0.001 for comparison). (**D**) Mice were scored weekly for arthritis and eye inflammation as described in Materials and Methods. After day 25, *P* < 0.001 for *B. melitensis* infected vs control mice at each time point and trajectory over time. (**E**) Front and hind paw widths were measured in millimeters (mm) with calipers, and the four paws were averaged for each mouse. The female infected group includes 14 mice. Changes in paw widths are vs days 14–18 of the experiment. After day 25, *P* < 0.001 comparing average paw widths for infected vs control mice at each time point and rate of change over time. End paw widths are the average per mouse on days 84–88 of the experiment. *P* < 0.001 vs control, with no difference between sexes. Circles represent individual mice. (**F**) Representative histologic sections from mice injected with PBS (top) or mice infected with *B. melitensis* (bottom) at 9.5 weeks post-infection. (**G**) Micro-CT images from mice injected with PBS (top) or mice infected with *B. melitensis* (bottom) at 12 weeks post-infection. ****P < 0.001.

Mice were infected intra-peritoneally with *B. melitensis*, as this route results in systemic dispersal of the bacteria mimicking human disease, including seeding of the spleen, liver, placenta, testes, and the skeleton (19). No effects of infection were observed until after 3 weeks, but by 4 weeks (“chronic” phase for mouse infection), 100% of the mice began developing arthritis in large weight-bearing joints (wrists and ankles/feet) with symmetric involvement (Fig. 1A and B). The hind paws exhibited mid-foot swelling reminiscent of tarsitis in human spondyloarthritis subjects. Small joints (paw digits) were spared. The second most prevalent manifestation was bilateral peri-ocular inflammation with an initial waxing and waning course, occurring after 5–6 weeks. Around 8 weeks, a few animals developed scaly rash on their paws. Tail swelling was rare. In the first experiment, a couple of control females developed mild late disease, but none developed in subsequent experiments. Infected animals also stopped gaining weight (Fig. 1C). Based on the two most common manifestations (eye disease and arthritis), mice were scored for clinical disease severity (Materials and Methods; Fig. 1D). Paw widths were also measured using calipers and revealed separation between infected animals and controls for end-experiment paw widths, as well as paw width changes over time (Fig. 1E). Clinical scores were less variable than the caliper measurements, effectively compressing variation, and the arthritis scoring correlated well with changes in caliper-measured paw widths (ρ ~ 0.76 and *P* < 0.001; Fig. S1) and end paw widths (ρ ~ 0.69 and *P* < 0.001; data not shown). There were no differences between males and females for clinical score or changes in paw width in response to infection with *B. melitensis*. Histologic sections of paws at 9.5 weeks post-infection showed extensive bony destruction, areas of florid fibrosis, foci of neutrophils resembling chronic abscesses, lymphocytic foci, as well as lymphocytic and macrophage infiltrates extending into the surrounding tendons and muscle (Fig. 1F). Although some mice developed transient diarrhea, gut histology by this point was normal. Strikingly, with the use of micro-computed tomography (CT) to visualize the skeleton, paws at 12 weeks exhibited both extensively moth-eaten bones adjacent to the wrist and exuberant new bone formation. New bone formation is a hallmark of human rheumatologic spondyloarthritis. Interestingly, the phalangeal and carpal bones were relatively spared (Fig. 1G).

### Clinical manifestations following infection with other *Brucella* species

**Fig 2:**
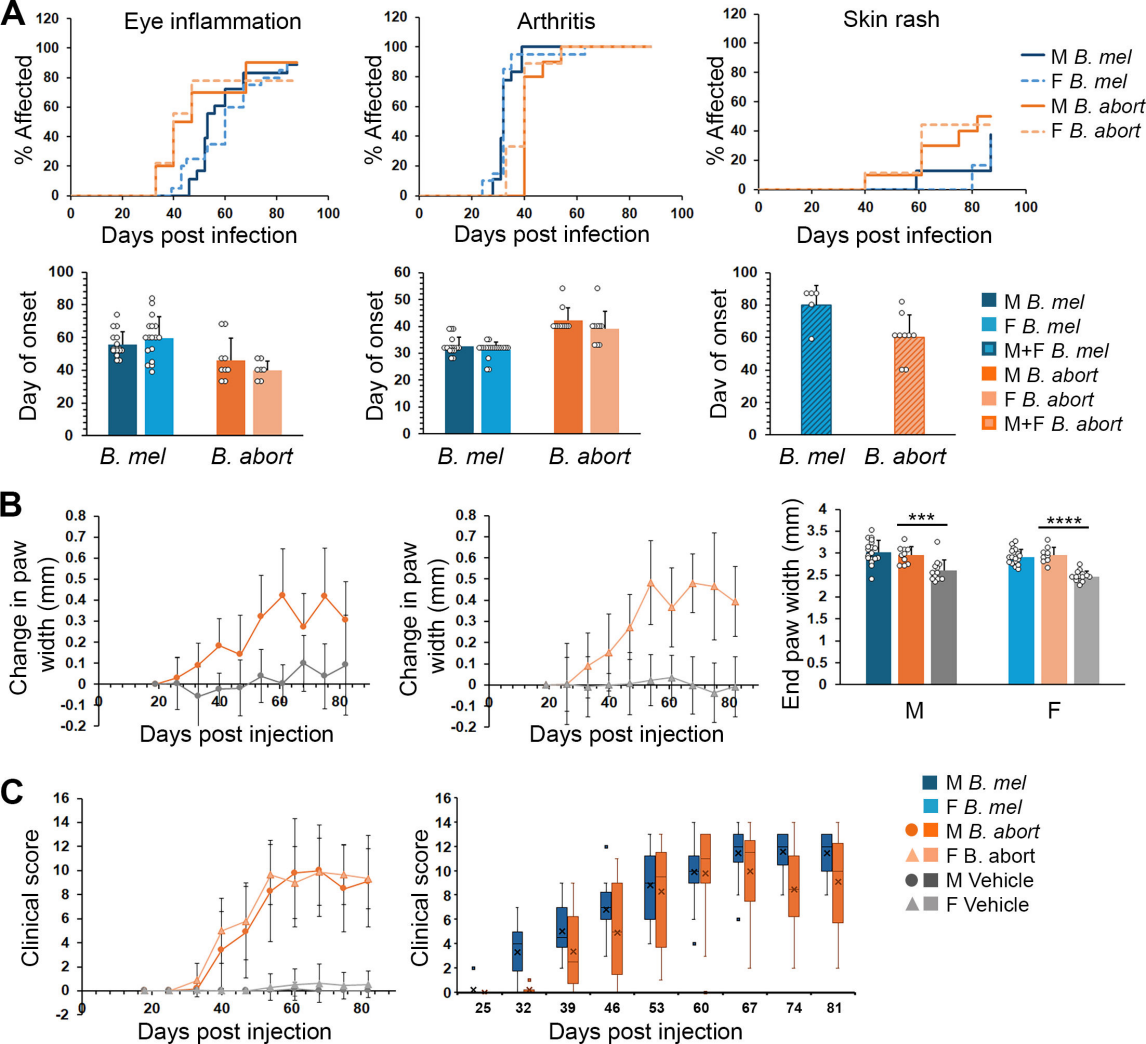
Comparison of diseases induced with *B. melitensis* and *B. abortus* in SKG mice. Mice were injected with PBS (vehicle control, *N* = 12 males, [M], and 13 females, [F]), 2 × 10^6^ *B. melitensis* 16M (*B. mel*, *N* = 18 males and 19 females), or 2 × 10^6^ *B. abortus* 2308 (*B. abort*, *N* = 10 males and 9 females) intra-peritoneally (i.p.). (**A**) Percent of mice (top row) that developed peri-ocular eye inflammation (left), arthritis (middle), and skin rash (right). Lower row is the median day of onset for each symptom. Skin rash values are for combined males and females related to low prevalence. Day of rash onset represents average from five *B. melitensis* and nine *B. abortus* mice. Error bars are SD. Circles represent individual mice. For arthritis onset, *P* < 0.001 comparing mice infected with *B. melitensis* and *B. abortus*. (**B**) Paw widths were measured by calipers, averaged as above, and are vs days 14–18 post-injection. Only infected animal paw widths significantly changed over time (*P* < 0.001). End paw widths are at day 81 and were significant for both *B. melitensis* and *B. abortus* vs control (*P* < 0.001). (**C**) Clinical scores over time in males and females infected with *B. abortus* (left) vs PBS vehicle control (*P* < 0.03 after day 25 for infected vs control animals). Right graph shows box-whisker plots with average, median, 25th and 75th percentiles, 10th and 90th (whiskers), and outlier points for males infected with *B. melitensis* vs *B. abortus*. ***P < 0.005; ****P < 0.001.

In comparing infection between *B. melitensis* 16M and *B. abortus* 2308, non-significant trends were noted in median onset of eye inflammation and paw rash (earlier onset, *P* = 0.11 and *P* = 0.06, respectively), but arthritis occurred about a week later with *B. abortus* (*P* < 0.001 for treatment effect; Fig. 2A). Changes in paw width diameters following *B. abortus* were significantly different vs. control PBS-injected animals (*P* < 0.001 for trends over time; Fig. 2B). End paw widths did not differ between *B. abortus* and *B. melitensis* but were greater than controls. Although *B. abortus* induced milder disease in some mice (see clinical score box-whisker plots for male mice infected with *B. melitensis* or *B. abortus*; Fig. 2C), the variation in clinical scores was not statistically significant comparing infection induced by these two *Brucella* species.

**Fig 3:**
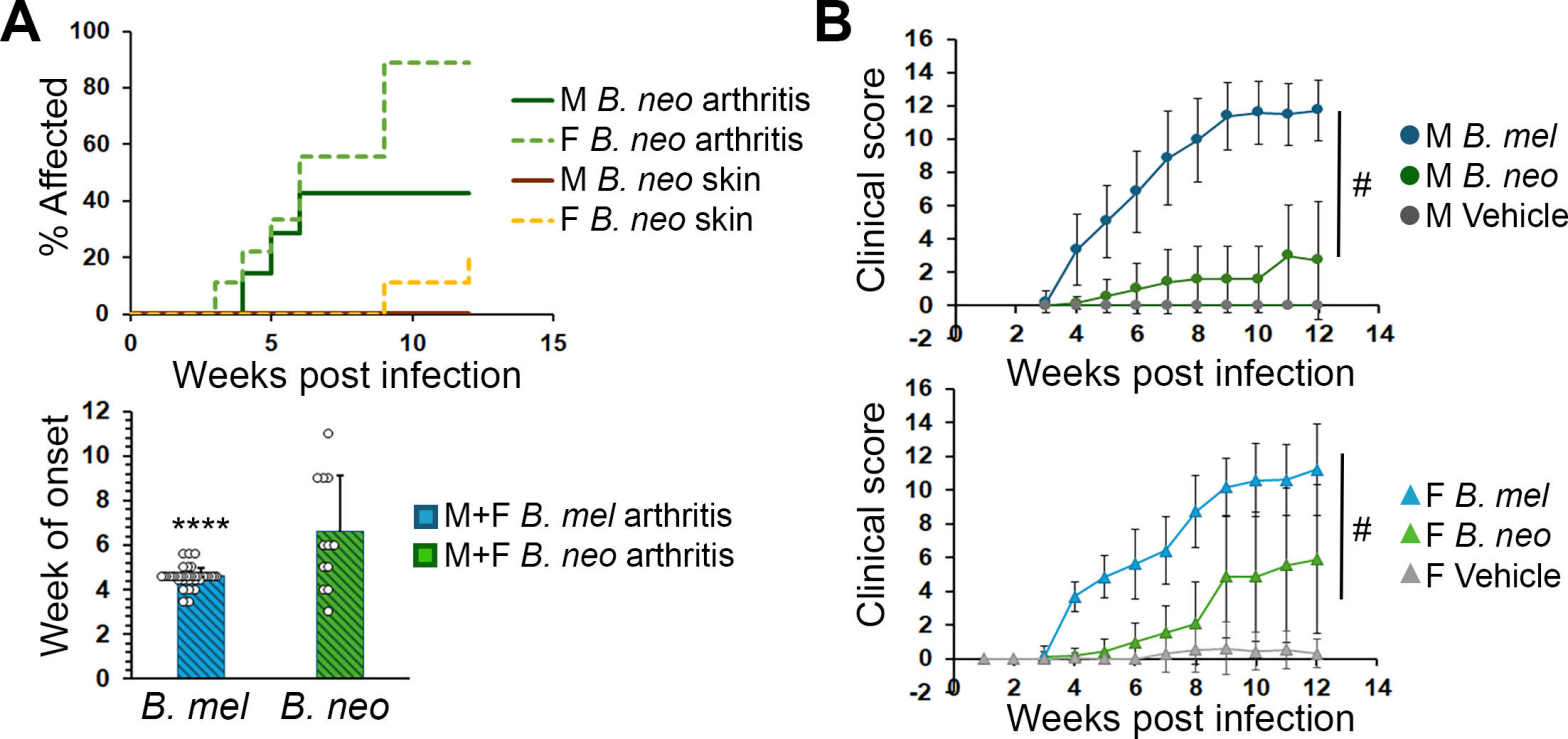
Later disease onset and milder clinical scores following *B. neotomae* (*B. neo*) infection. SKG mice were infected with 2 × 10^6^ *B. neotomae* or *B. neotomae* containing the lux operon (*N* = 7 males [M] and 9 females [F]).**(A**) Percent of mice affected by arthritis and skin rash (top) and comparison with *B. melitensis* for day of arthritis onset (bottom). (**B**) Clinical scores over time with comparison to *B. melitensis* and control. In both sexes, *B. neotomae* mice exhibited milder disease by week 4 as compared to animals infected with *B. melitensis* (*P* < 0.001 for pairwise comparisons and trends over time). Clinical scores of male infected with *B. neotomae* did not differ vs those of the controls. For females, *P* < 0.001 vs controls over time and at individual time points by week 9. **** and #, *P* < 0.001.

*B. neotomae*, a species found in wood rats and very rarely in humans (20), elicited an even milder disease and revealed sex differences: no peri-ocular inflammation was observed in any mice infected with *B. neotomae*. Arthritis penetrance was 40% in males vs 90% in females (*P* = 0.15). Arthritis onset was significantly delayed in mice infected with *B. neotomae*, as opposed to the more virulent *Brucella* strains (median onset of ~7.5 weeks; Fig. 3A; *P* < 0.001 vs *B. melitensis*). A couple of females developed paw skin rash, but no males developed. Changes in clinical score over time were significantly greater for female mice vs males (*P* < 0.001), although individual time point comparisons were not statistically significant due to variability. In comparisons with mice infected with *B. melitensis*, both *B. neotomae* males and females exhibited a milder disease (*P* < 0.001). In contrast to the females, clinical scores in males infected with *B. neotomae* did not differ significantly vs controls (Fig. 3B).

### Persistent *Brucella* infections in SKG spleen and paws

**Fig 4:**
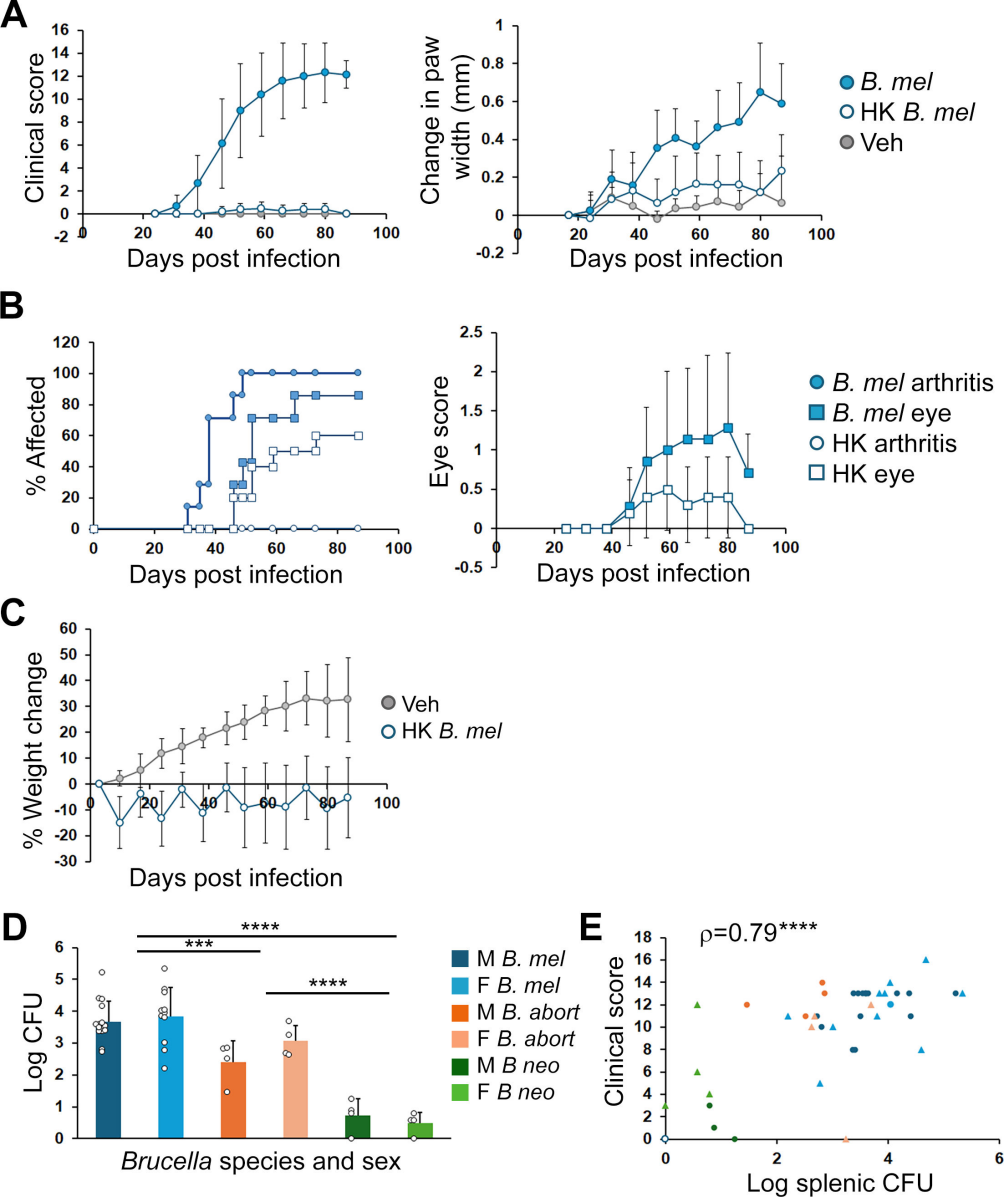
Contributions of persistent colonization to disease severity. (**A–C**) Mice were injected i.p. with PBS vehicle control (Veh, 4 mice), live *B. melitensis* (*B. mel*, 7 mice), or heat-killed *B. melitensis* (HK, 10 mice). Males and females were combined. (**A**) Joint and eye disease was scored over time (left) and changes in paw widths in millimeters (mm) vs 2 weeks (right). Pairwise comparisons of *B. melitensis* and heat-killed *Brucella* clinical scores were *P* < 0.03 by 4 weeks and change in score over time was *P* < 0.001. Paw widths for heat-killed *Brucella* changed significantly compared to both control animals and viable *B. melitensis* (*P* < 0.019 for all pairwise comparisons). (**B**) Percent of mice developing disease manifestations (left) and eye disease score (0–2; right). Accrual over time only differed for arthritis (*P* < 0.001). Average eye scores did not significantly differ. (**C**) Percent change in weight over time as compared to week 0. *P* < 0.001 for weight gain in controls only. (**D**) At 12 weeks following infection with *B. melitensis* (*B. mel*, M = 16 males, F = 11 females), *B. abortus* (*B. abort*, four each sex), *B. neotomae* (*B. neo*, four each sex), or heat-killed *Brucella* (10) splenic CFU was enumerated. Comparisons are between *Brucella* species treatment groups. (**E**) Correlation between final clinical score at 12 weeks and splenic CFU for the mice in panel **D**. For the *N* = 10 heat-killed *Brucella*, all CFU and final clinical scores were 0 (set to 0.1 for purposes of graphing). Spearman's rank correlation coefficient is 0.79, *P* < 0.001. ***P < 0.005; ****P < 0.001.

It was not clear if *Brucella*-induced arthritis in the SKG model is a purely “reactive process” to an infectious trigger or requires persistent infection. To further address this question, we used heat-killed *Brucella*. Infection with heat-killed *B. melitensis* did not induce obvious arthritis in comparison to live *Brucella*, although paw size subtly increased, suggesting the need for a viable infection rather than a simple pathogen-associated molecular pattern (PAMP) stimulation for significant arthritis (Fig. 4A). However, some mild peri-ocular inflammation was noted starting at day 46 (Fig. 4B) and there were no significant differences in the rate of eye disease accrual or score. Heat-killed mice treated with *B. melitensis* also failed to gain weight, exhibiting sawtooth fluctuations (Fig. 4C). To examine live, but persistence-defective *Brucella*, we used a *B. melitensis* mutant lacking the Type IV secretion system (T4SS) encoded by the VirB operon (Δ*virB*). Preliminary data with these mutants suggest a similar response to the heat-killed *Brucella* infection, with no significant arthritis and mild eye disease (Fig. S2). *Brucella* has been reported to persist in BALB/c spleens beyond 90 days, raising the question of whether the SKG mice were also impaired in clearance. In SKG mouse spleens harvested at 12 weeks, we observed differences between species, with *B. melitensis* showing the highest splenic CFU, *B. abortus* intermediate with about a log less, and *B. neotomae* with very low levels of splenic CFU (Fig. 4D). Spleens from mice administered with heat-killed *Brucella* and Δ*virB* were negative for CFU at the end of the experiment, as expected. Interestingly, splenic CFU from live *Brucella*-infected animals correlated moderately well with 12 weeks clinical score (ρ = 0.79; Fig. 4E), with this correlation mainly driven by differences in *Brucella* species and heat-killed *Brucella*, suggesting a relationship between the severity of systemic infection and arthritis.

**Fig 5:**
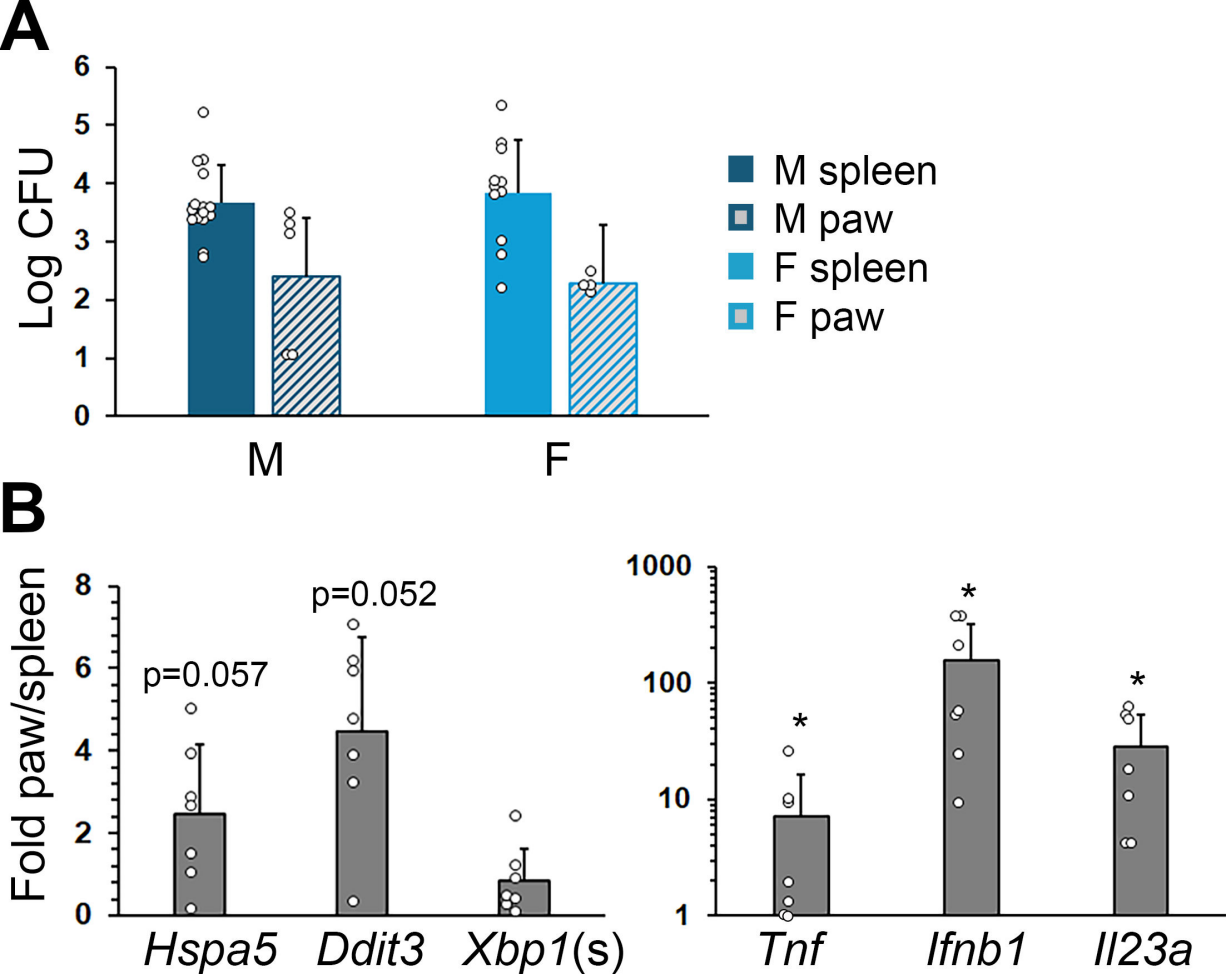
Paw CFU and gene expression. Male (M) or female (F) mice were infected with 2 × 10^6^ *B. melitensis* for 12 weeks prior to harvest of spleens and paw tissue. (**A**) Log CFU from spleens and paws. Results are from 15 male spleens, five male mouse paws, 11 female spleens, and four female mouse paws. (**B**) Spleens and paws were harvested for RNA, and gene expression was determined by quantitative PCR with normalization to the housekeeping gene 18S rRNA. Fold mRNA is the average ratio of paw to spleen gene expression within each mouse from three males and four females. Circles represent individual mice. *P < 0.05 for the ratio being different from 1.

Previous studies using *in vivo* imaging (IVIS) and our histology sections (Fig. 1F) suggested that *Brucella* colonizes the paws during infection (9). To determine if SKG paws contained viable *Brucella*, we dissected and evaluated the articular tissue for CFU. Paw tissue from mice infected with *B. melitensis* reliably grew detectable CFU at 12 weeks (Fig. 5A). Since the amount of tissue recovered from swollen paws was variable, we examined normalized gene expression for several Unfolded Protein Response (UPR) genes (*Hspa5*/BiP, *Ddit3*/CHOP, and spliced *Xbp1* [marker of active UPR]) and UPR-regulated cytokines (*Tnf*, *Ifnb1*, and *Il23a*) to gauge relative degree of infection. Indeed, normalized gene expression from paw tissue revealed greater levels of cytokines and a trend for *Hspa5* and *Ddit3*, suggesting that the paws had a relatively greater burden of infection compared to spleen (Fig. 5B).

### Use of the SKG model to probe attenuated *Brucella* mutants

**Fig 6:**
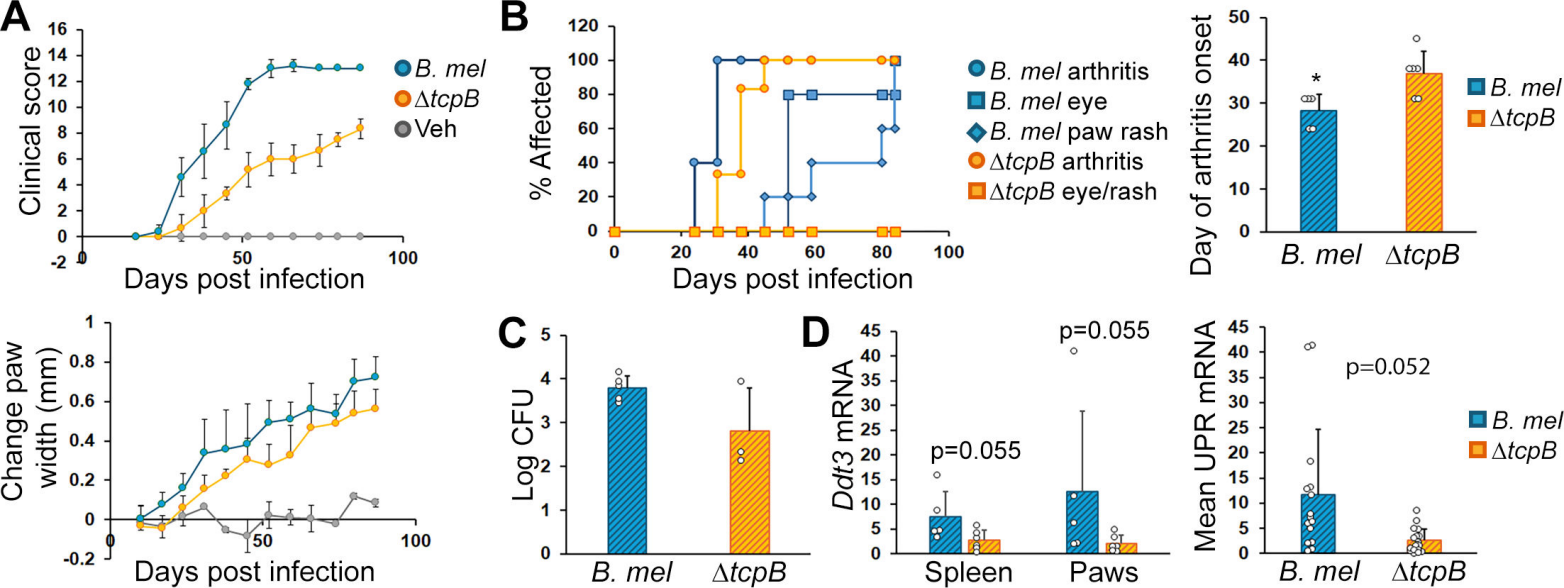
Milder disease with Δ*tcpB Brucella* mutant. Mice were infected i.p. with 2 × 10^6^ *B. melitensis* (*B. mel*, 3 males [M] and 2 females [F]), 2 × 10^6^ Δ*tcpB* mutant *B. melitensis* (3 M and 3 F), or PBS vehicle control (Veh, 1 M and 1 F). (**A**) Mice were scored weekly for 12 weeks. *B. mel* vs Δ*tcpB* over time and in pairwise comparisons after day 25, *P* < 0.001. Paw widths were measured by calipers weekly and averaged per mouse. All paw widths in infected mice change over time (*P* < 0.001), and comparisons of linear trends among wild type, mutant *Brucella,* and controls are all *P* < 0.002. (**B**) Percent of mice developing arthritis, peri-ocular eye inflammation, and psoriatic paw rash. Arthritis onset was later in Δ*tcpB* vs wild type, *P* = 0.02. (**C**) At 12 weeks, spleens were harvested, and *Brucella* was quantitated by colony-forming unit (CFU). CFUs are not significantly different. (**D**) Spleens and paws were harvested for RNA and mRNA quantitated by qPCR with normalization to 18S rRNA. Left is *Ddit3* expression, and the right is the combined averages of *Xpb1*(s), *Ddit3,* and *Hspa5* gene expressions in paw synovial tissue. Circles represent individual gene expression values.

The disease score differences seen with the various *Brucella* species suggested that this mouse model might be useful for evaluating the consequences of *Brucella* mutations for clinically relevant outcomes. The T4SS is required for *Brucella* secretion of effectors that modulate the macrophage intracellular milieu. One of these effectors, TcpB (also known as BtpA), has been reported to alter the inflammatory milieu *in vivo* without significant effect on colonization, providing another opportunity to assess the reactive component of disease. TcpB is also one of several *Brucella* effectors that support a pro-inflammatory endoplasmic reticulum response to the UPR. Mice infected with a TcpB deletion mutant (Δ*tcpB*) *B. melitensis* consistently displayed lower clinical scores throughout the period of 12 weeks as compared to the wild-type *B. melitensis*, as well as decreased paw width changes (Fig. 6A). Also, arthritis onset was a week later for the TcpB mutant-infected mice, and no TcpB mutant-infected mice developed peri-ocular inflammation or skin rash (Fig. 6). Splenic CFU was more variable in the ΔTcpB mutant-infected mice, but not statistically different from the parental *B. melitensis* wild type (Fig. 6). There was a non-significant trend toward decreased *Ddit3* (CHOP) expression in spleens and paws as well as average UPR gene expression in the paws of the Δ*tcpB* mutant-infected mice (Fig. 6).

## DISCUSSION

Herein, we describe a fully penetrant, easily scored clinical model of *Brucella*-induced spondyloarthritis in SKG mice with cardinal features of large joint arthritis, peri-ocular inflammation, and scaly paw rash. Except for some mild breakthrough diseases in two females when the colony was first getting established, non-infected controls have presented a clean comparator group. The model has enough dynamic range to reveal differences between wild type and mutant *Brucella*, as well as between different *Brucella* species. A more severe disease in females vs males infected with *B. neotomae* is intriguing. It is not yet clear whether this difference was revealed because of milder infection by *B. neotomae* (as evidenced by the lower splenic CFU vs *B. melitensis*) or whether this is a *Brucella* species-specific finding. The lack of eye and skin disease with higher dose *B. neotomae* supports the notion of species-specific inflammation. Results from the Δ*tcpB* and Δ*virB* mutants suggest that this model will be useful for investigating the genetic and molecular correlates of bacterial virulence *in vivo*.

The presence of splenic and paw CFUs at 12 weeks in mice infected with *B. melitensis* and *B. abortus* revealed active persistent infection. Moreover, during *Brucella* infection, there was a moderately positive correlation between clinical score and splenic CFU, particularly with the higher CFU infections. The absence of significant arthritic disease with heat-killed bacteria supports the idea that persistent infection is required for arthritis and suggests that PAMPs alone are not sufficient to elicit all disease manifestations. However, the mild eye inflammation and failure to gain weight suggest that the PAMP load is still having an adverse effect on the mice. On the other hand, the results from the *Brucella* mutant infections support the idea of a reactive component, as we confirmed that the ΔTcpB mutant consistently displayed an intermediate phenotype for most of the experiment duration without significant effects on splenic CFU.

It is not entirely clear whether SKG infection represents an apt model of human brucellosis or should be considered more of a non-specific infection-triggered spondyloarthritis model. The most common osteoarticular complications of brucellosis in humans consist of large joint arthritis (particularly knees), sacroiliitis, and spondylitis. Disease induced by the different *Brucella* species correlated somewhat with reported virulence in humans in that *B. melitensis* generally leads to the most prevalent and severe human infections, and *B. neotomae* rarely causes human disease—although *B. neotomae* has been rarely reported in human neurobrucellosis (21). The SKG mice did have a 3–4 week in developing overt signs of arthritis; thus, the onset of clinical disease is during the “chronic” phase of *Brucella* infection. In humans, osteoarticular disease takes several weeks to develop, with >60% occurring after 3 months. Also, up to 21% of humans with brucellosis develop ocular complications, most often uveitis. Interestingly, the prevalence of uveitis in ankylosing spondylitis is ~25%. Skin disease in humans with brucellosis is more uncommon, present in 1–14%. However, cutaneous involvement in humans is more often characterized by maculopapular skin eruption, rather than scaly psoriasis-like rash.

The phenotype of *Brucella*-infected mice differs from that of curdlan-injected mice in several ways. Onset of arthritis is rapid in curdlan-injected females, occurring 7 days post-injection. Male mice have delayed arthritis onset and less severe scores, although 100% eventually get arthritis by day 35. Curdlan-injected mice develop “dactylitis” or fusiform digital swelling, which was absent in our model. Only about 25% of curdlan-treated mice get eye disease, which consists of posterior uveitis and peri-ocular inflammation as seen in our model. Peri-ocular inflammation was more penetrant in our studies. Interestingly, new bone formation about the wrist is also seen following curdlan, along with severe erosions. A prominent feature of the curdlan-induced inflammatory disease is small bowel inflammation (ileitis) occurring in 50–60% by 10–12 weeks. In our model, there was no inflammation evident in the gut grossly or by histologic section. However, it is possible that we may not have looked far enough out, as experiments were ended at 12 weeks. Curdlan-driven disease is both gut microbiome and Th17 dependent.

Reports of *Chlamydia* infection in SKG mice suggest a different phenotype as well; thus, our *Brucella* results are not simply stereotypic SKG responses to any infection (18, 22). Male-female differences are more pronounced in this model than what we observed. Following genitourinary infection, 85% of females get arthritis, but male mice are spared. However, following intranasal infection, 50% of male mice get milder arthritis. Arthritis severity is asymmetric, unlike the experience with *Brucella*, where all paws were affected and to a similar extent. *Chlamydia*-infected mice also do not get dactylitis, as shown in our data. Compared to females, male mice infected with *Chlamydia* are much more prone to developing eye inflammation. Neither sex demonstrates ileitis. Disease severity correlated with infectious dose and degree of genitourinary inflammation. The authors suggested that reactive inflammatory disease resulted from insufficient sterilization of infection by SKG mice, as there was decreased IFN-γ and IL-17 productions, but excessive TNF-α.

Much work remains to establish the pathogenetic mechanisms underlying *Brucella*-induced spondyloarthritis disease in this robust new model. Moreover, the mechanisms may differ by site of involvement (e.g., eyes vs joint). On the host side, it will be interesting to determine how the altered T cell repertoire in SKG modulates control and anatomic distribution of *Brucella* as compared to the parental BALB/c strain. The host UPR has been implicated in rheumatologic spondyloarthritis. However, a recent report, wherein CHOP-deficient HLA-B27 transgenic rats actually developed similar or worse gut disease despite the lack of CHOP-dependent IL-23, suggests that the relationship between UPR and inflammation in spondyloarthritis will require further elucidation. *Brucella* induces a full UPR in infected macrophages; thus, this model presents an incredible opportunity for further dissection. The capacity to observe gradations in mouse disease severity as well as alterations in onset and sites of inflammatory involvement offers the potential for defining bacterial correlates contributing to virulence *in vivo*. In conclusion, the SKG mouse model should provide remarkable new opportunities for understanding the connections between *Brucella* infection and spondyloarthritis features.

## MATERIALS AND METHODS

### Zap70^SKG/SKG^ (SKG) mice and infection protocol

The SKG mouse line, first described by Sakaguchi and colleagues, carries a homozygous G-to-T substitution at nucleotide 489 in the *Zap70* gene on the BALB/c genetic background (23). SKG mice were kindly donated to our laboratory by Drs. David Riches and Elizabeth F. Redente of National Jewish Health, Denver, CO. The mice were rederived using pathogen-free recipients in the Genome Editing Facility of the University of Wisconsin-Madison (UW) Biomedical Research Model Services (BRMS). -For infection, *Brucella* was grown to late log to stationary phase, quantified, and diluted to 1 × 10^6^/100 µL in PBS. Each mouse was given 200 µL intra-peritoneally. Control animals received injections of vehicle (PBS) only. *Brucella* was heat-killed (HK) through incubation at 56°C for 1 hour.

### *Brucella* strains

All bacteria used in this study were cultured in brain heart infusion broth/agar (DOT Scientific). Antibiotics, when required, were added at the following concentrations: carbenicillin, 100 mg/L; kanamycin, 100 mg/L. Quantification of bacteria was from established growth curves through optical density measurements at 600 nm using a UV-visible spectrophotometer (Thermo Scientific). *Brucella* abortus S2308 and *Brucella melitensis* 16M were from University of Wisconsin archived stocks. *Brucella neotomae* was purchased from the American Type Culture Collection (ATCC). The *virB* operon knockout mutant of *B. melitensis* (Δ*virB*) was generated as previously described (24) using the plasmid pAVT1.4 (generously provided by Dr. Renee Tsolis). The *tcpB* (*btpA*) knockout mutant of *B. melitensis* (Δ*tcpB*) was generated as previously described (25). Bioluminescent *B. neotomae* expressing the luxCDABE operon was used in some experiments and was generated as previously described (9). All experiments with select agent *Brucella* strains were performed in a Biosafety Level 3 (or Biosafety Level 2 for *B. neotomae* only) facility in compliance with the Federal Select Agent Program (FSAP) in accordance with standard operating procedures approved by the University of Wisconsin-Madison Institutional Biosafety Committee.

### Clinical disease assessment

Arthritis assessment began 4 days after injections and continued weekly for 12 weeks. A thickness gage (Mitutoyo) was used to measure paw thickness at wrist joints of the front paws and ankle joints of hind paws. Measurements were recorded in millimeters. Paws were individually scored concordant with swelling and erythema, along with overall peri-ocular inflammation as follows: paw swelling of 0 (none), 1 (mild), 2 (moderate), 3 (severe); eye inflammation of 0 (none), 1 (mild), 2 (marked). Peri-ocular inflammation was typically bilateral. The clinical score was a sum of arthritis score in all four paws plus peri-ocular disease score (maximum of 14). For percent affected, the first day a symptom was observed was used for generating the Kaplan-Meier survival curves. Unscored observations that were recorded (yes/no) included diarrhea, tailbone swelling, rough skin, and scaly psoriatic paw rash. Mouse weight was recorded weekly.

### Spleen and paw synovia tissue isolation

Mice were euthanized by carbon dioxide and rinsed thoroughly with 100% ethanol. Spleens were placed in a 12 mL screw cap tube containing 3 mL MACS buffer (PBS, 2 mM EDTA, and 0.5% BSA) on ice. Mouse limbs were then dissected with scissors. Skin on each limb was peeled back and removed. Microsurgery scissors (FST 1502310) were used to cut remaining skin away from foot and paw. These scissors were then used to cut synovia from ankles, wrists, and paws, and synovia were placed in a MACS buffer-containing tube as with the spleens above. Synovia from all four limbs were combined in the same tube. Tissues were then dissociated using the gentleMACS system (Miltenybiotec) using C tubes and following the manufacturer's suggested protocol. After dissociation, tissue was divided equally (1.5 mL each) into two 1.7 mL microcentrifuge tubes. Samples were then pelleted in a microcentrifuge for 5 min at 400 × *g*. For the CFU assay, 1 mL lysis buffer (dH_2_O + 0.1% Triton X-100; Sigma) was added to one of the tubes and thoroughly vortexed (at least 20 seconds). For quantitative PCR (qPCR) assay, 0.5 mL RNAzol RT reagent (Molecular Research Center, Inc.) was added to the second tube and likewise thoroughly vortexed (at least 20 seconds). Samples for RNA isolation were then frozen at −80°C for later processing, while samples for CFU assay were processed immediately.

### Colony-forming units (CFU) and quantitative PCR (qPCR)

A modified most probable number (MPN) approach was used to enumerate *Brucella* in mouse spleen and synovia samples using 10-fold serial dilutions (26). Eight replicates were plated onto a square petri dish (100 mm × 100 mm) with grid (Fisher Scientific) containing BHI agar. CFU were enumerated after 4 days of growth.

Samples for total RNA isolation were thawed in RNAzol RT (MRC) and processed following the manufacturer's protocol. RNA quantity and quality were determined using a NanoDrop One spectrophotometer (Thermo Fisher). RNA was reverse transcribed using random primers (Superscript III; Invitrogen) following the manufacturer’s recommended protocol, and cDNA was quantified on a StepOnePlus Real-Time PCR System (ABI) normalized to 18S rRNA using the standard ΔCt/ΔCt method. The mouse primers used in this study were designed using IDT's online primer design tool and mixed with template in PowerUp SYBR Green master mix (Thermo Fisher) following the manufacturer's recommended protocol. Primer sequences were as follows: 18S rRNA—F, GGACACGGACAGGATTGACAG, and R, ATCGCTCCACCAACTAAGAACG; *Hspa5*—F, AGGATGCGGACATTGAAGAC, and R, AGGTGAAGATTCCAATTACATTCG; *Ddit3*—F, CATCACCTCCTGTCTGTCTC, and R, AGCCCTCTCCTGGTCTAC; *sXbp1*—F, GAGTCCGCAGCAGGTG, and R, GTGTCAGAGTCCATGGGA; *Ifnb1*—F, GGCATCAACTGACAGGTCTT, and R, ACTCATGAAGTACAACAGCTACG; *Il23a*—F, ACAAGGACTCAAGGACAACAG, and R, TGAAGATGTCAGAGTCAAGCAG; *Tnf*—F, TCTTTGAGATCCATGCCGTTG, and R, AGACCCTCACACTCAGATCA.

### Micro-computed tomography (CT)

*Ex vivo* µCT was performed at the University of Wisconsin-Madison Small Animal Imaging & Radiotherapy Facility (SAIRF) using the MILabs U-SPECT/CT^UHR^ (ultra high-resolution) system. Samples were prepared as follows: mice were euthanized, and limbs were dissected. Skin was removed from each limb. Limbs were then placed in 12 mL conical screw cap tubes (CELLTREAT) partially filled with EMA fixative (EtOH, methanol, acetic acid; 3:1:1). Then, gauze was inserted to keep the limb in place, and the tube was filled to the top and capped. After 24 hours, samples were scanned and images prepared by SAIRF staff (Dr. Justin Jeffries).

### Histological preparation and analyses

Sample preparation of front and hind paws for histological staining followed the standardized microscopic arthritis scoring of histological sections (SMASH) recommendations (27). Briefly, paws were cut 2–3 mm above the ankles or wrists, and the skin was removed. Samples were placed in 12 mL screw cap centrifuge tubes and filled with Decalcifier I (Surgipath) for fixation and decalcification. After 24 h, samples were sent to the Comparative Pathology Lab (CPL) of the UW-Madison where histology staff prepared H&E-stained slides. Slides were imaged and evaluated for arthritis by a pathologist (Dr. Thomas Warner of the Department of Pathology and Laboratory Medicine).

### Statistical analysis

Time to onset of disease manifestation was estimated using Kaplan-Meier methods and compared among treatment groups using log-rank tests. Poisson regression (with offset equal to the logarithm of follow-up time) was used to estimate occurrence rates for each treatment group and manifestation (eye, arthritis, skin), with cluster-robust standard errors used to account for multiple manifestations within the same mouse. Linear mixed-effect models were used to evaluate weight and paw width (change) over time. These models were also used to determine differences between treatment groups at individual time points, with *P* values adjusted for multiple comparisons to control the false discovery rate (FDR). Clinical scores were compared between groups of interest at individual time points using rank-based methods (Wilcoxon rank-sum test) and *P* values again adjusted (FDR method) for multiple testing. Scores were also summarized by computing the area under each animal's score profile over time; ordinary linear models were used to compare the resulting distributions of areas among groups of interest (defined by sex and/or treatment) or to determine whether there was a correlation (Spearman's ρ) with similarly computed areas over time for luminescence. Ordinary linear models were used to analyze (log-transformed) CFU abundance and UPR or cytokine gene expression for associations with treatment or sex. All *P* values were two-sided unless expressly stated. Analyses were performed using R (v.4.4.0; https://cran.r-project.org/). Error bars represent the means ± SD. Statistical significance is indicated in the figures (*P < 0.05, **P < 0.01, ***P < 0.005, ****P < 0.001) and figure legends.

## Data Availability

Requests for further information, resources, and reagents should be directed to and will be fulfilled by the corresponding author.

## SUPPLEMENTAL MATERIAL

Supplemental Figures mbio.00542-25-s0001.docx Figures S1 and S2.

ASM does not own the copyrights to Supplemental Material that may be linked to, or accessed through, an article. The authors have granted ASM a non-exclusive, world-wide license to publish the Supplemental Material files. Please contact the corresponding author directly for reuse.
