# Supplementary material for: Persistent articular infection and host reactive response contribute to Brucella-induced spondyloarthritis in SKG mice
Source: mBio. 2025 May 14;16(6):e00542-25. doi: 10.1128/mbio.00542-25 (PMC12153262; doi:10.1128/mbio.00542-25)
Supplement: Supplemental Figures — Figures S1 and S2. [file mbio.00542-25-s0001.docx]

**
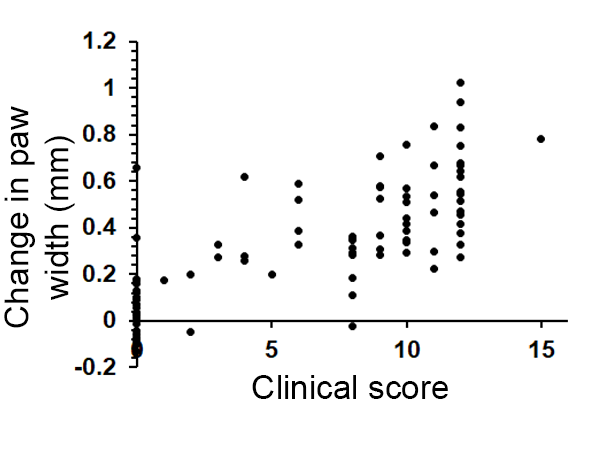
**

**Supplemental Figure S1: Correlation between paw width measurements and clinical scores**. Summary of mice infected with *B. melitensis, B. abortus, B. neotomae*, or injected with PBS vehicle control (N=39, 19, 16 and 25 respectively). Data points are end-experiment change in paw width vs. day 14-18 (in mm) and final clinical scores at days 81-88. Spearman’s correlation coefficient is ~ 0.76, p<0.001. There is also a correlation with final paw width (ρ ~ 0.69, p<0.001, not shown). The significance in correlation is driven largely by differences in infection treatment group.


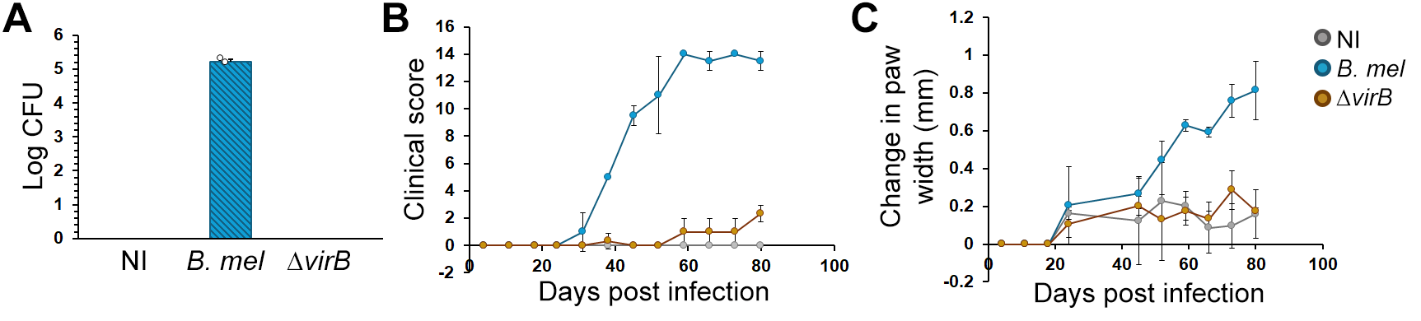


**Supplemental Figure S2: The Type IV secretion system is required for *Brucella* persistence and the development of arthritis.** Mice were injected with PBS (NI, non-infected, gray), wild type *B. melitensis* (*B. mel*, blue) or a mutant lacking the VirB operon encoding the Type IV secretion system (Δ*virB*, brown). Male and female mice were combined, with 3 Δ*virB* and 2 each of the *B. melitensis* and PBS controls. A) Splenic CFU at 12 weeks. B) Clinical scores over time. Note, the Δ*virB* mice developed mild peri-ocular inflammation but not arthritis. *B. melitensis* score trajectory was significant vs Δ*virB* (p<0.001), and Δ*virB* was not significant vs control (p=0.093). C) Changes in paw widths over time. Data points are on days of measurement by calipers and changes in millimeters are vs. day 18 (set=0). All animal paw widths increase over time (p<0.01). *B. melitensis* paw widths exhibit greater change vs. Δ*virB* over time (p<0.001). Δ*virB* paw width changes do not differ significantly from PBS controls.
